# Na^+^/K^+^-ATPase as a Target of Cardiac Glycosides for the Treatment of SARS-CoV-2 Infection

**DOI:** 10.3389/fphar.2021.624704

**Published:** 2021-04-15

**Authors:** Kauê Francisco Corrêa Souza e Souza, Bianca Portugal Tavares Moraes, Izabel Christina Nunes de Palmer Paixão, Patrícia Burth, Adriana Ribeiro Silva, Cassiano Felippe Gonçalves-de-Albuquerque

**Affiliations:** ^1^ Departamento de Biologia Celular e Molecular, Instituto de Biologia, Universidade Federal Fluminense, Niterói, Brazil; ^2^ Laboratório de Imunofarmacologia, Departamento de Bioquímica, Universidade Federal Do Estado Do Rio de Janeiro, Rio de Janeiro, Brazil; ^3^ Laboratório de Imunofarmacologia, Instituto Oswaldo Cruz, FIOCRUZ, Rio de Janeiro, Brazil; ^4^ Programa de Pós-Graduação Em Neurociências (PPGNEURO), Instituto de Biologia, Universidade Federal Fluminense, Niterói, Brazil; ^5^ Programa de Pós-Graduação Em Ciências e Biotecnologia (PPBI), Instituto de Biologia, Universidade Federal Fluminense, Niterói, Brazil; ^6^ Programa de Pós-Graduação Em Neûrologia/Neurociências, Hospital Antônio Pedro Universidade Federal Fluminense, Niterói, Brazil; ^7^ Programa de Pós-Graduação Em Biologia Celular e Molecular (PPGBMC), Universidade Federal Do Estado Do Rio de Janeiro, Rio de Janeiro, Brazil

**Keywords:** SARS-CoV-2, COVID-19, cardiac glycosides, Na+, K+ ATPase, molecular target, anti-viral, anti-inflammatory

## Abstract

Severe acute respiratory syndrome coronavirus 2 (SARS-CoV-2), identified for the first time in Wuhan, China, causes coronavirus disease 2019 (COVID-19), which moved from epidemic status to becoming a pandemic. Since its discovery in December 2019, there have been countless cases of mortality and morbidity due to this virus. Several compounds such as chloroquine, hydroxychloroquine, lopinavir-ritonavir, and remdesivir have been tested as potential therapies; however, no effective treatment is currently recommended by regulatory agencies. Some studies on respiratory non-enveloped viruses such as adenoviruses and rhinovirus and some respiratory enveloped viruses including human respiratory syncytial viruses, influenza A, parainfluenza, SARS-CoV, and SARS-CoV-2 have shown the antiviral activity of cardiac glycosides, correlating their effect with Na^+^/K^+^-ATPase (NKA) modulation. Cardiac glycosides are secondary metabolites used to treat patients with cardiac insufficiency because they are the most potent inotropic agents. The effects of cardiac glycosides on NKA are dependent on cell type, exposure time, and drug concentration. They may also cause blockage of Na^+^ and K^+^ ionic transport or trigger signaling pathways. The antiviral activity of cardiac glycosides is related to cell signaling activation through NKA inhibition. Nuclear factor kappa B (NFκB) seems to be an essential transcription factor for SARS-CoV-2 infection. NFκB inhibition by cardiac glycosides interferes directly with SARS-CoV-2 yield and inflammatory cytokine production. Interestingly, the antiviral effect of cardiac glycosides is associated with tyrosine kinase (Src) activation, and NFκB appears to be regulated by Src. Src is one of the main signaling targets of the NKA α-subunit, modulating other signaling factors that may also impair viral infection. These data suggest that Src-NFκB signaling modulated by NKA plays a crucial role in the inhibition of SARS-CoV-2 infection. Herein, we discuss the antiviral effects of cardiac glycosides on different respiratory viruses, SARS-CoV-2 pathology, cell signaling pathways, and NKA as a possible molecular target for the treatment of COVID-19.

## Introduction

Acute respiratory infections are the leading cause of morbidity and mortality from infectious diseases worldwide, due to highly contagious viruses and rapid dispersal, which may cause a collapse of the health system (Nair et al., 2011). In recent years, several viral epidemics have occurred, such as the severe acute respiratory syndrome coronavirus (SARS-CoV) epidemic in 2003, the influenza A H1N1 epidemic in 2009, and the Middle East respiratory syndrome coronavirus (MERS-CoV) epidemic in 2012. Severe acute respiratory syndrome coronavirus 2 (SARS-CoV-2), identified for the first time in 2019 in Wuhan, China, caused the coronavirus disease (COVID-19) that moved from epidemic status, to becoming a pandemic (Cascella et al., 2020). Several compounds have been proposed for COVID-19 treatment, but none have been shown to be effective. Although cardiac glycosides (CGs) are not specifically used for the treatment of viral infections (Buckalew, 2015), some studies have shown their antiviral effects on different respiratory viruses and associated this effect with the inhibition of Na^+^/K^+^-ATPase (NKA) (Sato and Muro, 1974). Herein, we discuss the antiviral activity of CG on respiratory viruses through NKA inhibition. We also suggest a role for NKA inhibitors as an option for COVID-19 therapy.

## CGs

CGs are a group of secondary metabolites that are widely distributed in nature, from different sources, and with different applications; however, they have a very similar chemical structure (Schonfeld et al., 1985). This consists of a steroid ring, a lactone ring with five or six carbons, and a sugar moiety (Prassas and Diamandis, 2008). These metabolites are divided into two subgroups: cardenolides, obtained from the extracts of *Digitalis*, *Strophanthus*, and the *Uregenia* plant species (Rodrigues-Mascarenhas et al., 2009) and bufadienolides, obtained mainly from toad toxins (Prassas and Diamandis, 2008; Ma et al., 2012). The broad diversity of these molecules is due to small differences in their structure (Prassas and Diamandis, 2008), which may influence the mechanisms by which these compounds disturb or inhibit DNA.

The effect of CG may involve Ca^+2^ calmodulin kinase (Diederich et al., 2017). Cardenolides are composed of a butyrolactone ring with five carbons, whereas bufadienolides contain a pyrone ring with six carbons (Prassas and Diamandis, 2008). Besides these structural differences, all CGs are C23 steroids with one or more sugar residues at C3 and a lactone ring at C17. Worldwide, digoxin is the cardenolide most used to treat patients with cardiac insufficiency, whereas ouabain is the most experimentally used. Both digoxin and ouabain act through NKA inhibition (Rose and Valdes, 1994) and are the most potent inotropic agents known (Buckalew, 2015). CGs do not promote disease regression but improve symptoms and stabilize clinical conditions (Abdul-Rahim et al., 2016).

## NKA

Na^+^/K^+^-ATPase is a transmembrane enzyme present in eukaryotic cells that maintains the electrochemical ion gradient between the extra- and intracellular medium by pumping two K^+^ ions into the cell and three Na^+^ ions out of the cell (Sen and Post, 1964; Buckalew, 2015). A classical inhibitor of NKA is known to be a CG (Rodrigues-Mascarenhas et al., 2009). Inhibition of NKA occurs through binding to the enzyme’s α subunit localized on the extracellular surface of the cytoplasmic membrane, blocking the enzyme in the phosphorylated mode E2 (Buckalew, 2015). Besides inhibitor ligand sites, the α subunit also contains ligand sites for K^+^ (extracellular surface) and Na^+^ (inner surface) (Mobasheri et al., 2000). The NKA also contains the β subunit, serving as a guide to stabilize the α subunit in the membrane and to regulate the affinity of the α subunit for K^+^ and CG (Mobasheri et al., 2000). The γ subunit modulates the affinity of the enzyme for different ligands (Therien et al., 2001) and has direct positive effects on the maximum velocity of ATP hydrolysis (Cortes et al., 2006).

The enzymatic inhibition was first correlated to the ionic transport of Na^+^ and K^+^ blockage, increasing intracellular Ca^+^ concentration and resulting in muscular contraction (Wasserstrom and Aistrup, 2005). Nevertheless, NKA inhibition by CG also triggers signaling pathways through the activation of protein kinase C (PKC), protein kinase A (PKA), phosphoinositide 3-kinase (PI3K) (Therien and Blostein, 2000), protein kinase B (AKT) (Yudowski et al., 2000), tyrosine kinase (Src) (Xie, 2003), nuclear factor kappa B (NFκB) (Kassardjian and Kreydiyyeh, 2008), and epidermal growth factor receptor (EGFR) (Li and Xie, 2009). This function seems to have been acquired through the incorporation of many domains that interact with proteins and ligands (Xie and Cai, 2003). According to Razani et al. (2002), the NKA type with a predominant function in cellular signaling is restricted to caveolae, areas of the plasma membrane with high levels of cholesterol, glycosphingolipids, and sphingomyelin. This is different to the type of NKA with an ion-pumping function, which is located along the membrane (Razani et al., 2002). The signal transduction function of NKA appears to be facilitated by properties that are independent of its function as an ion pump (Liang et al., 2007), because a low concentration of CG triggers cell signaling, and a high concentration causes the interruption of the ionic pump (Xie and Cai, 2003).

Several reports have shown the importance of ion pump-NKA alveolar epithelial cells for lung edema clearance (Olivera et al., 1994; Berthiaume et al., 1999; Borok et al., 2002). When the enzymatic activity is inhibited or increased, NKA plays a role in the clearance of edema (Sznajder et al., 2002). Factor et al. (1998) showed that the transfection of the α_1_-subunit and β1-subunit gene through adenovirus type 5 promoted overexpression of both subunits and increased the ion pump-NKA in the human lung cell line (A549), favoring pulmonary edema clearance. Additionally, active Na^+^ transport is important in the edema clearance of patients with acute respiratory illnesses (O’Brodovich, 1990; Sznajder et al., 2002). Different isoforms of NKA are expressed in different cell types. Alveolar cell type 1, which contributes to active Na^+^ transport, expresses NKA α2 (which contributes to alveolar fluid clearance), while NKA α1, the most ubiquitous, is expressed in alveolar cell type 1 and type II (Johnson et al., 2002; Ridge et al., 2003).

β-adrenergic compound agonists such as dopamine, terbutaline, and isoproterenol improve lung liquid clearance by upregulating NKA (Saldías et al., 2000; Saldías et al., 2002). At low concentrations, CG inhibits the enzyme, displaying cell signaling via NKA without interruption of ion-pump function, which is essential for edema clearance (Chioncel et al., 2015). The ion pump-independent action and signal transduction turn NKA into an important clinical treatment target.

## CGs as Anti-Inflammatory Drugs

CGs act on leukocytes that inhibit cell proliferation, migration, and the production of proinflammatory cytokines (Cavalcante-Silva et al., 2017; Furst et al., 2017; Cavalcante-Silva et al., 2020). CGs inhibit NFκB (Yang et al., 2005) and reduce viral entry into lung epithelial cells, directly decreasing proinflammatory cytokine production (Mahase, 2020). This is a prerequisite for viruses infecting lung epithelial cells and virus proliferation (Yang et al., 2005).

During lung epithelial cell infection, NFκB inhibition by the CG digitoxin abolished the production of proinflammatory cytokines, such as tumor necrosis factor alpha (TNFα), interleukin 1 beta (IL-1β), C-X-C motif ligand 1, growth-regulated oncogene/keratinocyte chemoattractant (GRO/KC), macrophage inflammatory protein 2-alpha (MIP2-alpha), monocyte chemoattractant protein 1 (MCP1), transforming growth factor beta (TGFβ), and interferon gamma (IFNγ) (Yang et al., 2005). Moreover, digitoxin inhibited hypersecretion of IL-8-dependent TNFα by blocking the recruitment of the TNF receptor-associated cell death domain protein (Srivastava et al., 2004; Yang et al., 2005).

*In vivo*, CG also has an anti-inflammatory role. Ouabain inhibits CD18 expression in monocytes; CD18 is an adhesion molecule involved in monocyte migration to the inflammatory site of injury (Cavalcante-Silva et al., 2020). Ouabain also reduces fluid extravasation, leukocyte infiltration, and the levels of the cytokines IL-1β and TNFα in mice with zymosan-induced peritonitis (Leite et al., 2015). Additionally, digoxin reduces the expression of IL-17, IL-1β, IL-6, TNFα, and IL-21 in mice with arthritis (Lee et al., 2015). Ouabain suppresses the production of IL-6 and TNFα by peripheral blood mononuclear cells that have been activated with lipopolysaccharide (LPS) from *E. coli*, protecting from LPS-induced lethality (Matsumori et al., 1997). The protective effect of ouabain was further studied in mouse acute lung injuries; ouabain decreased TNFα, IL-1β, and IL-6 production, diminished neutrophil and mononuclear influx, and reduced pulmonary permeability and edema formation (Wang et al., 2018). Furthermore, in a model of airway allergic inflammation, ouabain decreased the levels of IL-13 and IL-4 in bronchoalveolar lavage fluid, cell migration into peribronchiolar and perivascular areas, and mucus production in bronchioles (Galvao et al., 2017).

## CGs, NKA, and Respiratory Viruses

### Respiratory Viruses and NKA Expression and Activity

Balanian (1975) showed that NKA interactions with an adenovirus and another viral family, inhibited NKA enzymatic activity (Balanian, 1975). The interaction between some virus types and NKA subunits modulates enzyme activity, resulting in virus replication (Amarelle and Lecuona, 2018).

Some enveloped respiratory viruses downregulate the expression and activity of NKA (Table 1). The SARS-CoV envelope protein alters lung epithelium integrity and induces the displacement of basolateral NKA from the plasma membrane due to the bronchiolar barrier desquamation, impairing edema clearance by enzymatic activity (Nieto-Torres et al., 2015). Influenza A H1N1 (A/Hong Kong/54/98 strain) and H5N1 (A/Hong Kong/483/97 strain) strains affect alveolar fluid clearance by alveolar epithelial cells by NKA downregulation (Chan et al., 2016). Influenza A virus (IAV A/PR/8/34 strain) infection in alveolar epithelial cells also reduces NKA expression; it disturbs the host-signaling pathway by increasing the IFN-dependent TNF-related apoptosis-inducing ligand (TRAIL) (Peteranderl et al., 2016). The interaction of the H1N1-M2 protein (IAV A/PR/8/34 strain) with pulmonary cell NKA modifies the classic profile of the NKA α1 subunit’s basolateral expression and disperses the β1 subunit, worsening edema clearance during acute respiratory distress syndrome (ARDS)-induced by H1N1(IAV A/PR/8/34 strain) (Peteranderl et al., 2016). Decreased expression of the NKA α subunit has been observed in normal human bronchial epithelial cells infected with H1N1 (IAV A/Puerto Rico/8/1934^ΔGFP^ strain), resulting in pulmonary fluid homeostasis dysregulation (Brand et al., 2018). Table 1 shows the influence of respiratory viruses on NKA activity.

**TABLE 1:** Na^+^/K^+^-ATPase modulation by respiratory viruses.

Virus	NKA modulation	Modulatory effect	References
Non-enveloped respiratory viruses			
Adenovirus	Positive	Favors viral release into cytoplasm by lysis of endocytic vesicle	Seth et al. (1987)
Enveloped respiratory viruses			
Coronavirus	Negative	Difficult alveolar fluid clearance	Nieto-Torres et al. (2015)
Influenza A	Negative	Affects alveolar fluid clearance	Chan et al. (2016), Peteranderl et al. (2016)
Influenza A	Negative	Dysregulates pulmonary fluid homeostasis	Brand et al. (2018)
Human respiratory syncytial virus	Negative	Formation of macropinosomes, favoring viral entry	Lingemann et al. (2019)

The importance of NKA activity in viral replication has previously been reported. Our group has shown the importance of ion concentrations in the viral replication process. We have also observed that CG-induced changes in the concentration of Na^+^ and K^+^ interfere with the gene expression and virus yield of alphaviruses and inhibit their intracellular viral protein synthesis (Souza-Souza et al., 2020). This evidence corroborates the findings of Strauss et al. (1980), which showed the importance of ionic changes in host cells for replication of the Sindbis virus (mutant ts103), Semliki Forest virus, Middelburg virus, rhabdovirus, and vesicular stomatitis virus (Indiana serotype) (Strauss et al., 1980; Zhang et al., 2005).

Palù et al. showed that NKA activity was linked with several events after viral infection (Palu et al., 1994). Only infection by non-enveloped respiratory virus adenovirus (Ad2 strain) increases NKA activity during the replication process. The interaction between adenovirus and NKA increases enzymatic activity, favoring lysis of endocytic vesicles and resulting in the release of the virus into the cytoplasm. Nevertheless, it is unclear if the effect on the enzyme is direct or indirect (Seth et al., 1987).

### CG Activity Against Non-enveloped Respiratory Viruses

NKA inhibitors have been identified as a new antiviral therapeutic alternative. The antiviral activity of CG has been related to a variety of viruses, including adenovirus and rhinovirus A (Table 2). Adenovirus is known to cause a relatively mild upper respiratory tract disease and more severe bronchiolitis, pneumonia, diarrhea, meningoencephalitis, cystitis, and conjunctivitis (Rocholl et al., 2004; Chhabra et al., 2013).

**TABLE 2:** Antiviral mechanisms of cardiac glycosides.

Viruses	Cardiac glycosides	Antiviral mechanism	References
Enveloped respiratory viruses				
Influenza A	Ouabain, Adenium obesum	Inhibit viral replication	Amarelle et al. (2017) Kiyohara et al. (2012)
Human respiratory syncytial virus	Ouabain	Inhibits viral entry	Lingemann et al. (2019)
Middle East respiratory syndrome coronavirus	Ouabain, Bufalin	Inhibit early entry stage	Burkard et al. (2015)
Severe acute respiratory syndrome coronavirus 2	Digoxin, Ouabain	Inhibit viral replication	Cho et al. (2020)
Non-enveloped respiratory viruses				
Adenovirus	Digoxin, lanatoside C, ouabain, digitoxigenin, digitoxin	Alter RNA splicing, virucidal effect, RNA tranition	Stoilov et al. (2008) Grosso et al. (2017)
Rhinovirus A	Scillarenin 3-O-[N-(tert-butoxycarbonyl)-hydrazido] suberoyl)	Inhibit RNA synthesis, virucidal effect	Sato and Muro (1974) Kamano et al. (1988)

Stoilov et al. (2008) screened a library of known bioactive compounds for the capacity to modulate exon inclusion by microtubule-associated protein tau (MAPT) exon 10 in adenovirus mRNA. CGs (digoxin, lanatoside C, digitoxigenin, and ouabain) alter RNA splicing, interfering directly with viral translation (Stoilov et al., 2008). qRT-PCR analyses reveal that digoxin and digitoxin reduce genome levels at 20–22 h post-infection, altering RNA splicing of immediate-early protein (E1A) in the early stages of infection and partially blocking the RNA transition at late stages of adenovirus replication (strain HAdV-A31, -B35, -C5, and a species D conjunctivitis isolate) (Grosso et al., 2017). Additionally, digoxin and digitoxin have a virucidal effect between 2 and 4 h after adenovirus (HAdV-A31) interaction and reduce the yield of infectious progeny virions (Grosso et al., 2017).

Human rhinoviruses are the primary etiological agents of the ‘common cold’ (Skern et al., 1985). Although rhinovirus infections are self-limiting and present with mild symptoms, elderly patients, with or without respiratory disease, may have severe complications (Arruda et al., 1997). After the first step, i.e., attachment and penetration of the virus into the cellular cytoplasm, the viral RNA is translated into a single polyprotein that undergoes proteolytic self-cleavage by the viral proteases 2A and 3C (Skern et al., 1985). This generates functional structural and non-structural proteins (nsps) for continued viral RNA replication and assembly of progeny virions (Matthews et al., 1999). Sato and Muro (showed that scillarenin inhibits the picornavirus, especially strain 2060 of the rhinovirus, during RNA synthesis if added during the first half of the latent period (Sato and Muro, 1974). Kamano et al. analyzed 34 bufadienolides and two cardenolides in rhinovirus (2060)-infected HeLa cells (Kamano et al., 1988), finding that some bufadienolides exhibited better antiviral activity than cardenolides. In addition to scillarenin and 3-O-[N-(tert-butoxycarbonyl) hydrazide]suberoyl, both bufadienolides inactivated all viral particles (Sato and Muro, 1974). Table 2 contains some examples of the antiviral effects of CGs and their mechanisms of action.

### CG Activity Against Enveloped Respiratory Viruses

The inhibitory effects of CG occur at different phases of the life cycle of non-enveloped viruses and enveloped respiratory viruses such as influenza A, human respiratory syncytial virus (RSV), and coronavirus (Amarelle and Lecuona, 2018) (Table 2).

The influenza virus is a human pathogen causing annual epidemics that threaten to become worldwide pandemics due to the circulation of different virus strains (Potter, 2001). In 1918–1919, the influenza A H1N1 pandemic killed approximately 50 million people (Morens and Fauci, 2007). The most recent registered influenza pandemic was in 2009, causing approximately 18,500 confirmed deaths and affecting 214 countries (World-Health-Organization, 2009). The viral multiplication cycle starts with the influenza virus assembling sialic acid on the cellular glycoprotein or glycolipid through to the outer side of the HA molecule (de Graaf and Fouchier, 2014). Medical treatment of influenza, generally based on the administration of neuraminidase inhibitors (Oxford et al., 2002) is no longer effective. An *in vitro* screening model revealed that CGs have potential anti-influenza activity against strains A/WSN/33 (influenza A) and B/Yamagata/88 (influenza B) (Hoffmann et al., 2008). Amarelle et al. showed that 20 nM of ouabain, 50 nM of digoxin, and 100 nM of cinobufagin inhibit the influenza A virus. Ouabain inhibited influenza A replication between 4 and 6 h post-infection by decreasing intracellular K^+^ without impairing viral entry and mRNA transcription. Ouabain post-treatment did not inactivate influenza A particles (Amarelle et al., 2017). *Adenium obesum* (Forssk.) CG also inhibited influenza A virus (A/PR/8/34 strain) replication in Madin-Darby canine kidney cells at low concentrations (Kiyohara et al., 2012).

Parainfluenza viruses, the Sendai virus, frequently induce acute respiratory tract diseases in infants and immunocompromised adults (Glezen et al., 2000). The first step of the infection process is the attachment of the HN glycoprotein to its cellular receptor, followed by the release of a nucleocapsid structure containing the genome into the cytoplasm, then transcription of RNA in an mRNA species by RNA-dependent RNA polymerase (Xu et al., 2013). Nagai et al. (1972) reported that the action of ouabain on these viruses is time-dependent; treatment with ouabain is effective if added until 9 h post-infection of the Sendai virus (isolated from the Nagoya 1–60 strain), while pre-treatment with ouabain is not.

Human RSV causes severe lower respiratory tract infections in infants and young children worldwide (Lozano et al., 2012). The infection begins upon attachment of the virion to the apical surface of airway epithelial cells by the G glycoprotein (Zhang et al., 2002). After fusion, the helical ribonucleoprotein complex is released into the host cell cytoplasm (Rincheval et al., 2017). In the initial screening of 2560 compounds, only CGs significantly reduced RSV (rgRSV224 strain) infectivity, and digoxin and digitoxin showed antiviral activity with low cytotoxicity (Norris et al., 2018). A decrease in intracellular K^+^ inhibits RSV (rgRSV224 strain) replication. Furthermore, intracellular Na^+^ and K^+^ affect the viral multiplication cycle; changes occur in the initial 4 h of viral infection that interfere with viral RNA synthesis without decreasing attachment/entry steps (Norris et al., 2018). In another experiment, ouabain treatment led to reduced RSV yield (strain RSV-GFP), affecting an early infection step, such as viral entry (Lingemann et al., 2019).

**FIGURE 1:**
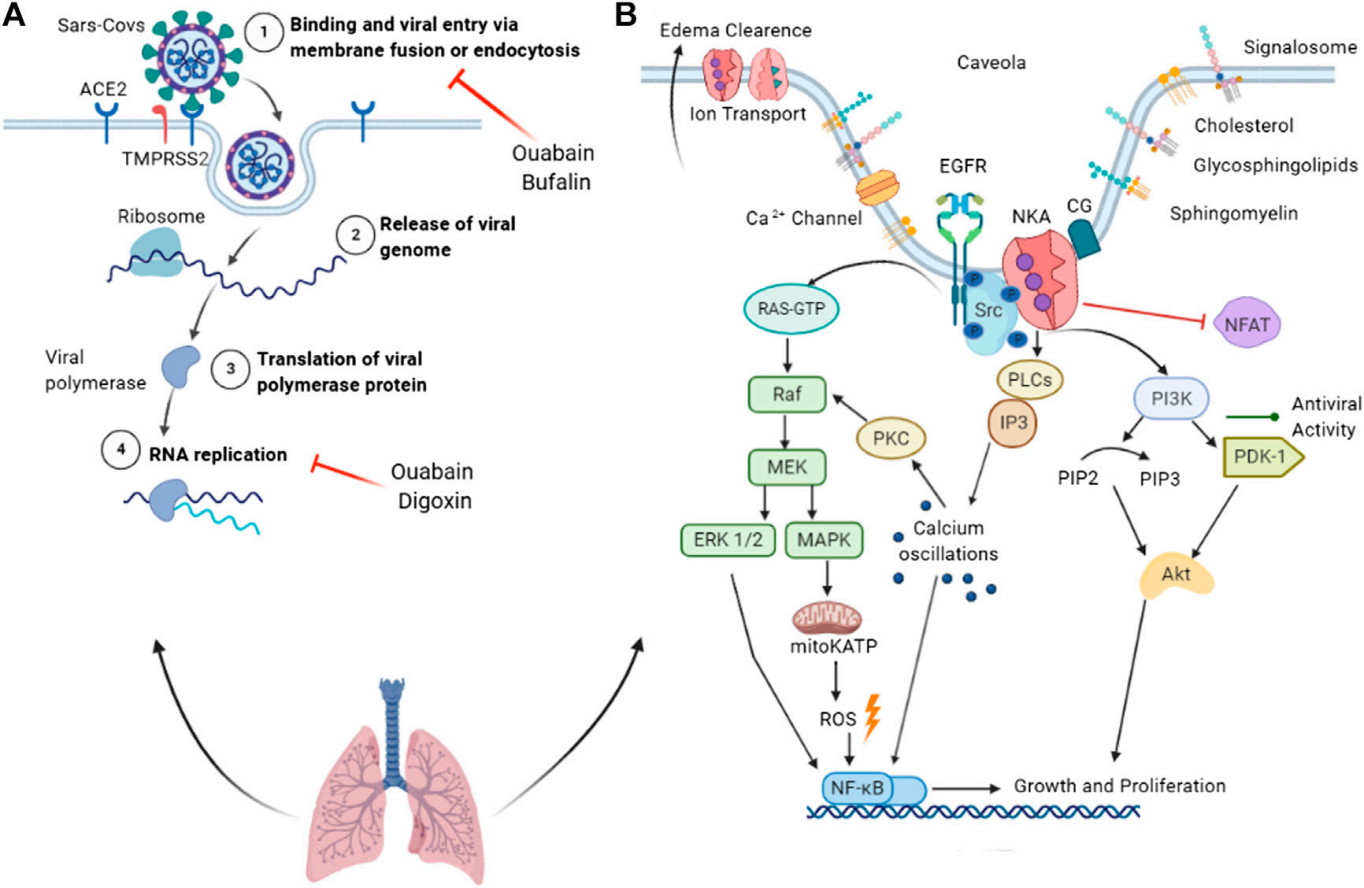
Summary of the antiviral properties of cardiac glycosides and Na+/K+-ATPase as a signal transducer. **(A)** Binding of SARS-CoV spike protein to the ACE2 receptor leads to membrane fusion or endocytosis, a process inhibited by ouabain and bufalin (1). Once in the cytoplasm, the viral genome is released (2) and translated into replicase proteins. The polyproteins are cleaved by a virus protease into individual replicase complex nonstructural proteins (nsps) (3), forming the replication-transcription complexes where replication begins (4). **(B)** Na+/K+-ATPase acts in ion transport, edema clearance, and as a signal transducer. Cardiac glycoside binding to the preassembled Na+/K+-ATPase (pump) signalosome in caveolae transduces signals via multiple pathways. Activated NKA rapidly activates Src tyrosine kinase, which activates the EGFR. Activated EGFR recruits protein adaptors that activate the Ras-GTP complex, leading to MEK pathway activation. MAPK activation triggers the opening of mitochondrial ATP-sensitive potassium channels (mitoKATP), resulting in ROS production and NFκB activation. The MEK pathway also activates NFκB through ERK ½ activation. In parallel, Src modulates the activation of the PI3K/PDK1 pathway, which is associated with viral activity and replication suppression. Activated PI3K phosphorylates Akt, which phosphorylates a variety of downstream pathways related to growth, survival, and proliferation. NKA activates phospholipase C (PLC) and inositol-1,4,5-triphosphate (IP3); the latter binds to the IP3 receptor of the endoplasmic reticulum, releasing calcium ions into the cytoplasm. Calcium oscillation activates PKC and NFκB. ACE2, angiotensin-converting enzyme 2; TMPRSS2, transmembrane protease serine 2; Src, non-receptor tyrosine kinases; EGFR, epithelial growth factor receptor; PKC, protein kinase C; PI3K, phosphoinositide 3′ kinase; PLC, phospholipase C; MAPK, mitogen-activated protein kinase; MEK, MAPK–ERK activating kinase; ROS, reactive oxygen species; mitoKATP, mitochondrial ATP-sensitive potassium channel.

Burkard et al. (2015) showed that ouabain and bufalin inhibit MERS-CoV infection by silencing or inhibiting the NKA α1-subunit at an early entry stage. Ouabain shows potent inhibitory effects against transmissible gastroenteritis coronavirus (TGEV), with IC50 values ranging from 143 ± 13 nM, decreasing the number of viral RNA copies (Yang et al., 2017; Yang et al., 2018). CGs also inhibit SARS-CoV-2 infection; ouabain and digitoxin inhibit viral mRNA expression, copy number, and viral protein expression of SARS-CoV-2 at the post-entry stage (Hoehl et al., 2020) (Figure 1A).

### CGs and Coronaviruses

#### Epidemiology of SARS-CoV-2 Infection

Recently, SARS-CoV-2 was discovered in China and spread rapidly worldwide with greater virulence than the SARS-CoV outbreak in 2003 (Huang et al., 2020). Coronavirus infection can cause gastroenteritis, hepatitis, respiratory tract infections resembling the common cold, lower respiratory tract infections such as bronchitis and pneumonia, SARS, systemic diseases, and even death in humans (Owusu et al., 2014).

Since the first report of SARS-CoV-2 infection in December 2019 from the World Health Organization (WHO) Country Office in China, the number of COVID-19 cases has been growing steadily at an alarming pace (WHO, 2020b). According to the WHO, the cases identified outside of China in the early global spread occurred because infected travelers traveled abroad. Countries such as Australia, Canada, Cambodia, France, Finland, Germany, India, Italy, Japan, Nepal, Malaysia, the Philippines, the Republic of Korea, Singapore, Sri Lanka, Thailand, the United States of America (USA), United Arab Emirates, and Vietnam were the first to report Chinese traveler-associated COVID-19 cases (World-Health-Organization, 2020b). Some months after the initial report in China, the first SARS-CoV-2 infection notifications in South America emerged; Brazil, Peru, and Ecuador were the countries with the most significant number of cases (Dong et al., 2020). Currently, the USA and Brazil are the epicenters of the COVID-19 pandemic (World-Health-Organization, 2020a).

Data from China reveal that SARS-CoV-2 is highly virulent compared to SARS (Li Q. et al., 2020). The incubation period for SARS-CoV-2 is 4.5–5.8 days (Lauer et al., 2020). The infection then evolves to a pre-symptomatic stage (1–3 days) through symptomatic infection (2–4 weeks) to a prolonged post-symptomatic or recovery stage (2–8 weeks) (Subbarao and Mahanty, 2020). The median age of hospitalized patients with COVID-19 was 63 years (Bordallo et al., 2020). The SARS-CoV-2 infection affects people who are 2–72 years of age, with a high prevalence in men (Backer et al., 2020). People with chronic cardiovascular and pulmonary disease, immunodeficiency, hypertension (Ejaz et al., 2020; Flaherty et al., 2020), and diabetes, along with elderly patients (Wang et al., 2020) are more prone to infection and death by SARS-CoV-2 than healthy people. In Brazil, chronic heart disease is the deadliest comorbidity in COVID-19 patients (Pachiega et al., 2020).

The fatality rate tends to vary, ranging from 2.8 to 11% (Wang et al., 2020) with a median of 5% (Li L. Q. et al., 2020). There have been more than 15 million cases across the world and more than 625,000 people have died (World-Health-Organization, 2020c). However, sub-notification and delays to case confirmations can quickly change the statistics (Rocha-Filho, 2020).

The recovery time for COVID-19 is approximately 14 days (Noor et al., 2020; Wang et al., 2020). Asymptomatic SARS-CoV-2-infected people and patients in incubation or those who have recovered from COVID-19 may shed infectious virus particles (Hoehl et al., 2020) for up to 3 or 4 weeks (Lan et al., 2020), representing a considerable challenge for disease prevention and control.

SARS-CoV-2 has a broad spectrum of infection from asymptomatic patients to patients who develop mild, moderate, or severe forms of ARDS (Vardhana and Wolchok, 2020). Most patients have mild symptomatology without needing hospital treatment, with the appearance of fever, a dry cough, and tiredness. Less common symptoms are headache, aches and pains, nasal congestion, diarrhea, sore throat, loss of taste, or loss of smell (Struyf et al., 2020).

#### Pathogenesis of SARS-CoV-2 Infection

SARS-CoV-2 is transmitted predominantly *via* respiratory droplets and direct contact (Lai et al., 2020). SARS-CoV enters the host cell through interaction between the viral spike protein and the angiotensin-converting enzyme 2 (ACE2) of the host cell, triggering the virus's fusion with the cellular membrane and consequently releasing the viral genome into the cytoplasm (Belouzard et al., 2009). In the cytoplasm, replicase gene translation ensues from the virion genomic RNA, which encodes two large open reading frames: rep1a and rep1b. These express the polyproteins pp1a and pp1ab, posteriorly translated in nsps (Brierley et al., 1989).

As with SARS-CoV, the SARS-CoV-2 virus enters cells by binding its spike to ACE2 (Hoffmann et al., 2020). After this first contact, SARS-CoV-2 uses an enzyme, the cellular serine protease TMPRSS2, to prime the spike protein (Hoffmann et al., 2020). The spike protein of SARS-CoV-2 contains a receptor-binding domain (RBD) that specifically recognizes ACE2 as its receptor. The SARS-CoV-2 RBD contains a core and a receptor-binding motif that mediates tight binding with ACE2 (Shang et al., 2020). The C-terminal domain (CTD) of SARS-CoV-2 also binds to ACE2. Wang et al. (2020) demonstrated that key residue substitutions in the SARS-CoV-2 CTD slightly strengthen the interaction with ACE2 and leads to a higher affinity for receptor binding than with the SARS RBD (Wang et al., 2020). This interaction between the S protein and its receptor is responsible for the species specificity and tissue tropism of the virus (Li et al., 2005). The cleavage of the viral protein by TMPRSS2 is crucial for the fusion between the virus and cellular membrane, starting the viral infection process (Hoffmann et al., 2020). Some cells show accentuated vulnerability to infection, such as type II alveolar cells, myocardial cells, esophagus epithelial cells, proximal tubule cells of the kidney and ileum, and urothelial bladder cells (Zou et al., 2020).

The SARS-CoV-2 S-protein sequence contains 12 additional nucleotides upstream of the single Arg cleavage site 1, which is associated with a canonical furin-like cleavage site (Coutard et al., 2020). This furin-like cleavage site may support virus egress by being cleaved for S-protein priming and may provide an advantage in transmission when compared to other lineages of beta-coronaviruses (Coutard et al., 2020). Coronavirus replication occurs entirely in the cytoplasm (Subbarao and Mahanty, 2020). After entry and uncoating, the virus genome is translated into the pp1a and pp1ab replicase polyproteins to start RNA synthesis in the replicase–transcriptase complex (de Wilde et al., 2018). Viral RNA synthesis produces genomic and subgenomic RNAs, and the viral structural proteins are inserted into the endoplasmic reticulum, where mature virions are formed (Fehr and Perlman, 2015).

Higher expression of ACE2 may prolong the virus multiplication cycle, enhance virus replication, and mediate penetration of the virus into the host cell (Tian et al., 2020). SARS-CoV and SARS-CoV-2 bind to ACE2, leading to downregulation (Kumar et al., 2020). The virus appears to enter cells *via* the membrane receptor, which is functionally removed from the external site of the membrane, consequently increasing the bioavailability of angiotensin 2 (Verdecchia et al., 2020). Angiotensin 2 is associated with other pulmonary diseases such as ARDS by triggering significant inflammatory lesions in the respiratory tree-like inflammatory infiltrate, edema, and alveolar wall thickening, which contribute to heightened viral pathogenesis (Imai et al., 2005; Kuba et al., 2005).

In the lungs, over-activation of angiotensin II receptor type 1 (Ang2-AT1R) leads to severe lung injury and lung failure due to pneumonia (Banu et al., 2020). Activation of the Ang2-AT1R pathway activates NFκB, increasing the expression of proinflammatory cytokines (Albini et al., 2020). It also activates the JAK/STAT pathway and ADAM17, leading to downstream production and release of IL-6 (Catanzaro et al., 2020) and inactivating ACE2 (Eguchi et al., 2018), respectively. Additionally, the Ang2-AT1R pathway mediates the conversion of a soluble form of IL-6 (Murakami et al., 2019) and is vasoconstrictive and pro-fibrotic (Albini et al., 2020; Banu et al., 2020), playing an essential role in the pathogenesis of COVID-19. The vasoconstrictive effect caused by the increase in angiotensin 2 and subsequent dysfunction of the renin-angiotensin-aldosterone system could be the reason some patients have shallow blood oxygen levels but are not breathless (Subbarao and Mahanty, 2020). One of the suggested mechanisms behind this is that oxygen uptake is obstructed because of congested and constricted blood vessels in the lungs, but not due to accumulation of edema fluid in the alveoli (Subbarao and Mahanty, 2020). The most severe symptom observed in COVID-19 patients is interstitial pulmonary edema, which is common in 90% of cases (Lu et al., 2020). Some studies have shown that this clinical manifestation is supported by ACE2 downregulation, which increases alveolar capillary permeability and leads to interstitial and alveolar edema (Imai et al., 2005).

ACE2 expression is mainly associated with innate and acquired immune responses: regulation of B cell-mediated immunity, secretion of the cytokines IL-1, IL-10, TNFα, IL- 6, and IL-8, and activation of neutrophils, NK cells, and T cells (Costela-Ruiz et al., 2020). ACE-2 downregulation induces macrophage activation syndrome (MAS) (Banu et al., 2020), which is characterized by uncontrolled activation and proliferation of T lymphocytes and macrophages, leading to a cytokine storm and multiple organ failure (Bracaglia et al., 2017).

One of the leading causes of death from COVID-19 is a cytokine storm or cytokine release syndrome, which is also associated with ARDS (Wang et al., 2020). Severe cases tend to demonstrate lymphocytopenia and a higher leukocyte count (Vabret et al., 2020). Recognition of SARS-CoV-2 activates downstream transduction pathways such as NFκB, JAK-STAT, and IRF3, leading to a large release of inflammatory cytokines, such as IL-6, IL-1β, IL-2, IL-8, IL-17, CCL3, and TNFα; such cytokines are increased in severely ill patients (Catanzaro et al., 2020). The expression of proinflammatory genes, such as chemokines, are elevated in COVID-19 patients compared to in community-acquired pneumonia patients and healthy controls, causing chemokine-dominant hypercytokinemia (Zhou et al., 2020).

During infection of host cells by viruses, IFN-stimulated genes (ISGs) and type I IFNs are expressed to induce an antiviral state (Subbarao and Mahanty, 2020). IFN-stimulated genes (ISGs) are increased in COVID-19 patients; however, compared to other viruses, SARS-CoV infections induced fewer ISGs. In addition, SARS-CoV-2 may have developed mechanisms to delay the IFN response by inhibiting innate immune signaling (Zhou et al., 2020).

Inflammatory cytokine IL-6 levels are significantly elevated in critically ill patients' serum; this is related to the need for mechanical ventilation and mortality (Zhou et al., 2020). This cytokine's consistent elevation might function as a predictive biomarker for disease severity (Gao et al., 2020). High IL-6 levels amplify the innate immune system response by inducing the release of more cytokines, heightening the influx of neutrophils (Hunter and Jones, 2015), activating downstream JAK/STAT3 and MAPK pathways, and subsequently activating several genes involved in inflammation and immunity (Garbers et al., 2015; Jordan et al., 2017).

Beyond immune cells, IL-6 causes endothelial cell activation and dysfunction by inducing the expression of chemoattractant proteins and adhesion molecules that recruit immune cells into the sub-intimal space (Qu et al., 2014). IL-6 also induces excessive vascular endothelial growth factor (VEGF) production, leading to enhanced angiogenesis (Tanaka et al., 2014). The effects induced by IL-6 are associated with cardiovascular diseases; several studies have already reported IL-6 alterations in such diseases and atherosclerosis (Qu et al., 2014; Wainstein et al., 2017; Zhang et al., 2018).

There is a high prevalence of cardiovascular diseases among fatal cases of COVID-19 (Clerkin et al., 2020). The most common manifestations are acute myocardial injury and arrhythmias (Bansal, 2020). Myocardial injury is mainly manifested as an increase in high-sensitivity cardiac troponin I levels (>28 pg/ml) (Huang et al., 2020). The increase in some inflammatory biomarkers (IL-6, ferritin, lactate dehydrogenase, and D-dimer) concomitantly raises the possibility of this being associated with the cytokine storm. Angiotensin II activates JAK/STAT (Schieffer et al., 2000) and the NFκB pathway in COVID-19 patients (Kranzhofer et al., 1999), increasing IL-6 production. Another mechanism is the myocardial dysfunction from the direct effect of SARS-CoV-2 on the heart, mediated by ACE2 (Clerkin et al., 2020).

**FIGURE 2:**
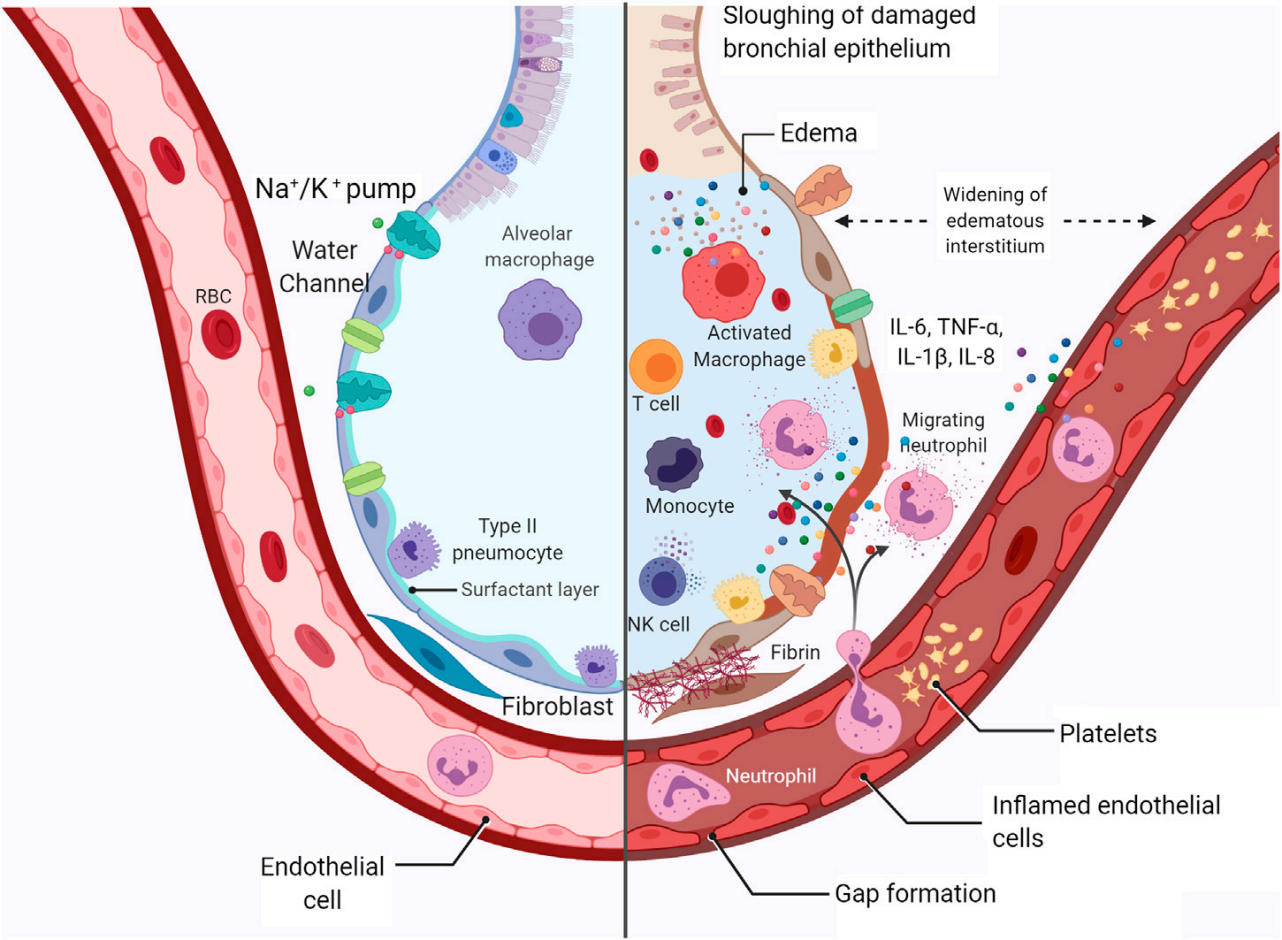
Representation of healthy alveoli and SARS-CoV-2-injured alveoli. Representation of alveolar damage during COVID-19 infection, with interstitial and alveolar edema, cytokine activation, neutrophil migration, Na^+^/K^+^-ATPase, water channel assisting the edema removal, diapedesis, recruitment of T cells and NK cells, epithelial cell death, and initial fibrin deposition. Created with BioRender.com.

Another striking characteristic of SARS-CoV-2 infection is the high incidence of interstitial pulmonary edema and pulmonary alveolar edema (Figure 2) (Tian et al., 2020), that is, leakage of fluid from pulmonary capillaries into the interstitium and alveoli. Lu et al. (2020) demonstrated that 90% of COVID-19 patients showed interstitial pulmonary edema in the lungs. Some pulmonary edema mechanisms may include ACE2 downregulation with increased alveolar-capillary permeability, leading to interstitial and alveolar edema (Imai et al., 2005).

The enzyme NKA plays a pivotal role in edema clearance in the lungs (Goncalves-de-Albuquerque et al., 2015). Active transport of Na^+^ by basolateral NKA drives the transepithelial fluid from the airspaces to the capillaries, improving edema clearance (Gonçalves-de-Albuquerque et al., 2016). The decreased expression of NKA results in the accumulation of pulmonary fluid (Zhang et al., 2018). Activation of NKA is also linked to lung inflammation, as reported by our group (Goncalves-de-Albuquerque et al., 2012; Goncalves-de-Albuquerque et al., 2013; Goncalves-de-Albuquerque et al., 2014). The SARS-CoV infection has already been shown to hamper pulmonary edema clearance by NKA due to the flaking of the bronchiolar barrier, resulting in the displacement of the enzyme (Nieto-Torres et al., 2015).

#### CG Use in COVID-19 Treatment?

So far, there are some vaccines in the final test phase (Sharma et al., 2020). Among these, the sputnik V has already been widely used in Russia to vaccinate doctors and teachers (Logunov et al., 2020). Several drugs have also been suggested to treat patients infected with SARS-CoV-2 (Elfiky, 2020; Hossain et al., 2020), including chloroquine and its analogue, hydroxychloroquine. However, some studies have shown that these antimalarial drugs can cause adverse reactions, such as skin changes, neuromyopathy (Estes et al., 1987), cardiotoxicity (Mahon et al., 2004), dysfunction of lysosomal enzymes that leads to impairment of intracellular degradation processes and accumulation of glycogen and phospholipids (Homewood et al., 1972) and retinopathy (Marmor et al., 2011). Moreover, *in vitro* tests using chloroquine and hydroxychloroquine showed that these compounds did not decrease acute SARS-CoV-2 infection (Touret and De Lamballerie, 2020). *In vivo* tests have also shown that chloroquine and hydroxychloroquine are not effective in inhibiting SARS-CoV replication in a mouse model (Barnard et al., 2006). Other potential compounds include lopinavir–ritonavir; however, a study in hospitalized adult COVID-19 patients showed that treatment with these drugs did not significantly improve clinical improvement or decrease mortality and viral RNA detection (Cao et al., 2020). Remdesivir is effective against SARS, MERS, and SARS-CoV-2 *in vitro*; clinical studies show promising results, but they are still under investigation (Chatterjee, 2020).

Other prophylactic/therapeutic options should be explored, including human monoclonal antibodies, IFNs, and siRNAs, and low-molecular-weight SARS-CoV inhibitors targeted at any of the specific processes involved in the viral replication cycle (such as viral entry into the cells, proteolytic cleavage, RNA replication, and transcription) (De Clercq and Field, 2006). In this review, we have discussed the effect of CGs on respiratory viruses; these compounds inhibit viral entry and replication without inducing cell death. Additionally, they inhibit NKA, impairing Na^+^ and K^+^ movement (Buckalew, 2015). Intracellular transport of molecules and ions (Boulant et al., 2015), such as K^+^ (Hartley et al., 1993) and Na^+^ (Hoffmann et al., 2008) is essential for the viral replication process, making NKA a possible molecular target and CG an anti-SARS-CoV-2 drug candidate.

Some studies have shown the efficacy and safety of CGs. Digoxin use reduced recurrent hospitalizations to treat congestive heart failure (CHF) and decreased the severity of CHF (Digitalis Investigation Group, 1997). Digoxin decreased all-cause 30-day hospital admissions in older patients (mean age, 72 years) with chronic systolic CHF. Kaur et al. (2009) reported a significant reduction in cerebral infarct size and prevention of ischemia/reperfusion-induced cognitive and motor deficits after digoxin treatment. An *in vitro* study has also shown the inhibition of prostate cancer cell proliferation by digoxin, digitoxin, and ouabain without damaging normal cells (Yeh et al., 2001).

A low concentration of CG triggers Src signaling via NKA, resulting in the inhibition of SARS-CoV infection (Figure 1B) (Burkard et al., 2015). Src modulates PI3K-AKT signaling (Liu et al., 2007) and is involved in ouabain-induced Ca^2+^ oscillation by affecting the interaction between the IP3 receptor and α1 NKA (Fontana et al., 2013). The activation of NKA-dependent PI3K-PDK1 signaling by ouabain is an essential contribution to anti-transmissible gastroenteritis coronavirus activity (Yang et al., 2018). Protein kinase C also regulates NKA affinity by compounds (Ridge et al., 2003). The interaction of ouabain and NKA also activates a cellular signaling cascade involving Src, mitoKATP, and reactive oxygen species (ROS). In this pathway, ouabain protects outer mitochondrial membrane integrity, adenine nucleotide compartmentation, and energy transfer efficiency, boosting the myocardium’s resistance to oxygen deficiency (Pasdois et al., 2007). In addition, Src activation by CG can trigger MAPK extracellular signal-regulated kinase 1/2 signaling through the Raf-MEK cascade, causing effects independent of alterations to intracellular calcium (Wong et al., 2018). Nimmerjahn et al. (2004) demonstrated the importance of NFκB for influenza virus (A/FPV/Bratislava (H7N7) and A/WSN/33 (H1N1) strains) infection in human cells, reporting that cells with low NFκB activity are resistant to influenza virus infection and a normalization increase in NFκB activity suppresses viral infection. In a mouse model of SARS-CoV infection, blockage of NFκB activation prevented cytokine storms, resulting in increased survival (DeDiego et al., 2014). Digoxin blocks the host cytokine storm induced by influenza (A/Wuhan/H3N2/359/95 strain), inhibiting NFκB and nuclear factor of activated T-cells (NFAT). Inhibition of NFκB and NFAT blocks TNFα, GRO/KC, MCP1, MIP2, IL-1β, TGFβ, and IFNγ, decreasing the inflammatory process and consequently improving the infection outcome. Ouabain and digitoxin show antiviral activity, inhibiting mRNA and viral protein expression of SARS-CoV-2 (Cho et al., 2020). Based on these data, CGs can be considered anti-SARS-CoV-2 drug candidates.

## Final Remarks

CGs are steroidal compounds known to inhibit NKA activity by binding to the enzyme’s α subunit. Initially, it was believed that the inhibitory effect on NKA would lead to changes in the ionic gradient; however, it was discovered that CGs also trigger cell signaling activation through NKA inhibition. The first use of a CG in medicine focused on treating patients with heart failure. This is because CGs are the most potent inotropic agents; this feature is characterized by the activation of the Na^+^/Ca^++^ exchanger, responsible for removing intracellular Na^+^ accumulation through exchange with Ca^++^ and favoring muscle contraction. Many biological activities have been assigned to CGs, including antitumor, anti-inflammatory, molluscicide, pro-apoptotic, and antiviral activities. The latter has raised interest because some viral infections such as SARS-CoV-2 do not have specific treatments or vaccines.

Some drugs such as chloroquine, hydroxychloroquine, lopinavir-ritonavir, and remdesivir have been tested as candidates for alternative COVID-19 therapies. Research has shown the antiviral activity of ouabain, bufalin, and digitoxin on MERS-CoV and TGEV (Burkard et al., 2015), while ouabain and digitoxin have antiviral effects against SARS-CoV-2 (Hoehl et al., 2020). The mechanism of action of CG on coronaviruses may involve cell signaling activation by NKA inhibition. SARS-CoV-2 infection activates NFκB, and digoxin inhibits NFκB and inflammatory cytokine production, directly interfering with viral yield. This evidence is supported by a study showing higher resistance to infection by the influenza virus (A/FPV/Bratislava (H7N7) and A/WSN/33 (H1N1) strains) in cells with low NFκB activity (Nimmerjahn et al., 2004). NFκB appears to be regulated by Src (Kang et al., 2006) and NFκB activation can occur due to the alteration of intracellular Ca^+^ from Src-EGFR-RAS regulation (Li and Xie, 2009). The antiviral effect of CGs may therefore be associated with Src activation (Burkard et al., 2015).

The antiviral activity of CGs appears to be related to the activation of cell signaling through NKA inhibition. Because CG use is based on its antiviral and anti-inflammatory effects, it combines two strategies: decreasing viral replication and negatively modulating the exacerbated inflammatory response observed in COVID-19 patients. Depending on the dose, CGs can induce different effects, activating signaling without drastically affecting NKA activity and, therefore, the ionic gradient. Thus, further studies are required to determine the effect of NKA inhibitors on SARS-CoV-2 infection.
